# Prognostic Association Between Perioperative Red Blood Cell Transfusion and Postoperative Cardiac Surgery Outcomes

**DOI:** 10.3389/fcvm.2021.730492

**Published:** 2021-09-24

**Authors:** Yanxiu Li, Iokfai Cheang, Zhongwen Zhang, Xiangrong Zuo, Quan Cao, Jinghang Li

**Affiliations:** ^1^Department of Critical Care Medicine, The First Affiliated Hospital of Nanjing Medical University, Nanjing, China; ^2^Department of Cardiology, The First Affiliated Hospital of Nanjing Medical University, Nanjing, China; ^3^Department of Hepatobiliary and Pancreatic Surgery, The Affiliated Jiangning Hospital of Nanjing Medical University, Nanjing, China; ^4^Department of Cardiovascular Surgery, The First Affiliated Hospital of Nanjing Medical University, Nanjing, China

**Keywords:** cardiac surgery, MIMIC-III, perioperative, red blood cell transfusion, outcomes

## Abstract

**Objective:** To investigate the correlation between red blood cell transfusion and clinical outcome in patients after cardiac surgery.

**Methods:** Demographic, clinical characteristics, treatment with/without transfusion, and outcomes of patients after cardiac surgery from the Medical Information Mart for Intensive Care—III database were collected. Patients were divided into two groups according to perioperative transfusion. A multivariable logistic regression analysis was utilized to adjust for the effect of red blood cell transfusion on outcomes for baseline and covariates and to determine its association with outcomes.

**Results:** In total, 6,752 patients who underwent cardiac surgery were enrolled for the analysis. Among them, 2,760 (40.9%) patients received a perioperative transfusion. Compared with patients without red blood cell transfusion, transfused patients demonstrated worse outcomes in inhospital mortality, 1-year mortality, and all-cause mortality. Adjusting odds ratios (ORs) for the significant characteristic, patients with perioperative transfusion remained significantly associated with an increased risk of inhospital mortality [OR = 2.8, 95% confidence interval (CI) 1.5–5.1, *P* = 0.001], 1-year mortality (OR = 2.0, 95% CI 1.4–2.7, *P* < 0.001), and long-term mortality (OR = 2.2, 95% CI 1.8–2.8, *P* < 0.001).

**Conclusion:** Perioperative red blood cell transfusion is associated with a worse prognosis of cardiac surgery patients. Optimal perioperative management and restricted transfusion strategy might be considered in selected patients.

## Introduction

Anemia is a common morbidity in cardiac surgery patients due to systemic malnutrition, malabsorption, inflammation, inadequate erythropoiesis, and/or chronic blood loss (1). Studies have demonstrated that preoperative anemia is an independent risk factor for postoperative morbidity and mortality (2, 3).

Cardiac surgery is associated with perioperative blood loss and a high risk of allogeneic blood transfusion (4). The incidence of perioperative blood transfusion is dependent on the complexity and duration of surgery, prevalence of preexisting anemia, and age of the patient (5). Cardiac surgery often requires the support of cardiopulmonary bypass (CPB), which can significantly induce an inflammatory response and has been associated with perioperative anemia (6, 7). Therefore, red blood cell (RBC) transfusions are usually inevitable during these procedures.

Blood transfusions are generally considered safe. Although transfusions are considered lifesaving treatments, as they provide immediate improvement of anemia, they do not address the underlying cause. Also, certain risks of transfusion reactions, including allergic reactions, acute immune hemolytic reaction, delayed hemolytic reaction, graft-vs. host disease, etc., would affect patients' outcomes as well (8). In post-cardiac surgery patients, previous studies suggested that both preoperative anemia and RBC transfusion are associated with inhospital mortality and have a strong impact on long-term survival (9, 10). These might be related to the overlap immune reaction and the systemic perioperative condition.

Given the existing knowledge, the present study aimed to further explore the characteristics and the association of RBC transfusion with mortality and adverse events in cardiac surgery patients from the Medical Information Mart for Intensive Care (MIMIC)—III database.

## Methods and Material

### Study Population and Design

The current large single-center retrospective study used publicly available data from the MIMIC-III database conducted by Beth Israel Deaconess Medical Center (BIDMC, Boston, MA, USA). Further details on MIMIC-III protocols are available from its original publication (11).

Demographics, procedures, vital signs, laboratory examination results, input and output information, medications, and other clinical variables were collected for further analysis. Adult patients admitted to the intensive care unit from 2001 to 2012 were included in the initial analysis. Among the 46,520 patients in the full sample, after excluding patients age <18 years old, those with a lack of complete blood counts recorded within 24 h ICU admission, and >5% missing variables, there were a total of 6,752 patients enrolled in the present study.

### Baseline Variables and Definitions

Data were extracted by structured query language PostgreSQL 9.6. Demographic variables such as age, gender, and comorbidity (hypertension, chronic heart failure [CHF], diabetes mellitus, chronic obstructive pulmonary diseases, chronic kidney disease, and liver dysfunction) were obtained.

Vital signs, blood gas analysis (pH, base excess, anion gap, bicarbonate, and lactate), laboratory results, mechanical ventilation time, and perioperative transfusion were collected. Body mass index (BMI, kilograms/meter squared) was calculated as body weight divided by height squared. The sequential organ failure assessment (SOFA) score (12) and systemic inflammatory response system (SIRS) score (13) at admission to the ICU were calculated. Transfusion of packed RBCs was calculated as binary 0 (no transfusion) or 1 (transfusion given) in the dataset.

The primary outcomes were inhospital mortality, 1-year mortality, and long-term all-cause mortality. The secondary outcomes were the length of ICU stay, length of hospital stay, and mechanical ventilation time. The follow-up time was defined as the period between admission time (ADMITTIME) and date of death (DOD_SNN) in the MIMIC-III database. To be noted, the long-term follow-up was continued until death or December 2016 for the all-cause death after a 1-year follow-up, whichever occurred first.

### Statistical Analysis

Continuous variables are presented as means ± standard deviations for normal distribution data and median ± interquartile range for non-normal distribution data. Categorical variables are presented as numbers (%). Comparison analyses were conducted using the Mann–Whitney *U*-test for continuous variables and the χ^2^ test or Fisher exact test for categorical variables.

Univariable logistic regression analysis was conducted to estimate the relationships between perioperative transfusion and all outcomes. Multivariable logistic regression analysis was then performed to adjust for the following significant covariates. Cox regression analyses were performed to determine whether the perioperative transfusion affected the mortality and other study outcomes. The effect was expressed by odds ratio (OR) or hazard ratio (HR) with a 95% confidence interval (CI). Adjustment with the variables associated with primary and secondary outcomes was performed. These include the age, sex, BMI, comorbidity (hypertension, chronic heart failure, and chronic kidney disease), blood gas results (pH value, base excess, anion gap, bicarbonate, and lactate), laboratory tests (white blood cell count, platelets count, hemoglobin (HB), serum creatinine (SCr), and international normalized ratio), SOFA score, and SIRS score.

Statistical analysis was performed using STATA version 15 (STATA Corp LLC, College Station, TX, USA) and SPSS version 26.0 (IBM, Armonk, NY, USA). *P* < 0.05 was considered statistically significant.

## Results

### Baseline Characteristic

A total of 6,752 cardiac surgery patients (median age 67 years old, 68.4% male) were included. The median follow-up time was 1,717.83 (927.81, 2,520.21) days, with the longest follow-up time of 10 years.

According to treatment with or without RBC transfusion during hospitalization, patients were divided into two groups (non-transfusion group and perioperative transfusion group). Among them, 2,760 (40.9%) patients received blood transfusion perioperatively, and the median amount of RBC transfusion for the transfusion group was 750 (375, 1,483.75) ml. The characteristics are compared in Table 1.

**Table 1:** Baseline characteristics.

**Characteristic**	**Without transfusion** ***N* = 3,992**	**Perioperative transfusion** ***N* = 2,760**	***P***
Age, years	65.5 ± 21.1	69.3 ± 12.1	<0.001
Male, *n* (%)	3,016, 75.6%	1,672, 60.6%	<0.001
BMI, kg/m^2^	29.0 ± 5.9	28.0 ± 5.8	<0.001
Comorbidity			
HTN, *n* (%)	2,886, 73.2%	1,886, 68.3%	<0.001
CHF, *n* (%)	933, 23.4%	1,034,37.5%	<0.001
DM, *n* (%)	1,278, 32%	938, 34%	0.092
COPD, *n* (%)	590, 14.8%	419, 15.2%	0.652
CKD, *n* (%)	307, 7.7%	326, 11.8%	<0.001
Liver dysfunction, *n* (%)	84, 2.1%	57, 2.1%	0.931
Blood gas analysis			
Ph	7.31 ± 0.05	7.30 ± 0.07	<0.001
BE	−2.9 ± 2.6	−3.8 ± 3.4	<0.001
AG	12.2 ± 2.7	13.2 ± 3.3	<0.001
Bicarbonate	25.0 ± 2.4	24.5 ± 2.9	<0.001
Lactate	2.7 ± 1.3	3.6 ± 2.4	<0.001
Laboratory tests			
WBC, × 10^9^/L	14.9 ± 5.7	14.5 ± 6.4	0.006
Platelets, × 10^9^/L	131.5 ± 44.6	113.6 ± 48.6	<0.001
Hemoglobin, g/dl	12.9 ± 1.5	12.1 ± 1.5	<0.001
SCr, mg/dl	1.0 ± 0.7	1.2 ± 1.2	<0.001
INR	1.5 ± 0.5	1.7 ± 0.8	<0.001
SOFA score	4.5 ± 2.4	5.4 ± 2.7	<0.001
SIRS sc ore	2.9 ± 0.9	3.0 ± 0.9	<0.001
Outcomes			
Ventilation time > 48 h, *n* (%)	138, 3.7%	520, 19.2%	<0.001
Hospital Stay > 14 days, *n* (%)	380, 9.5%	708, 25.8%	<0.001
ICU stay > 3 days, *n* (%)	875, 22.4%	1,541, 58.2%	<0.001
Inhospital mortality, *n* (%)	30, 0.8%	93, 3.4%	<0.001
1-year mortality, *n* (%)	114, 2.9%	229, 8.3%	<0.001
Long-term mortality, *n* (%)	403, 10.1%	694, 20.1%	<0.001

*BMI, body mass index; HTN, hypertension; CHF, chronic heart failure; DM, diabetes mellitus; COPD, chronic obstructive pulmonary diseases; CKD, chronic kidney disease; BE, base excess; AG, anion gap; WBC, white blood cell; SCr, serum creatinine; INR, international normalized ratio; SOFA, sequential organ failure assessment; SIRS, systemic inflammatory response system*.

Age, gender, BMI, comorbidity of hypertension, CHF, chronic kidney disease, blood gas indexes, white blood cell count, platelets count, HB, SCr, international normalized ratio (INR), SOFA score, SIRS score, and study outcomes were all significantly different between the groups (*P* < 0.05 in all cases).

### Primary Analysis

To determine whether perioperative transfusion was associated with the prognosis of cardiac surgery patients, univariate and multivariate logistic regression analyses (Table 2) were performed.

**Table 2:** Logistic regression model of the perioperative red blood cell transfusion and postoperative outcomes.

**Outcomes**	**Univariate**			**Multivariate^#^**		
	**OR**	**95% CI**	***P***	**OR**	**95% CI**	***P***
Ventilation time > 48 h, *n* (%)	6.2	5.1–7.5	<0.001	3.3	2.5–4.3	<0.001
Hospital Stay > 14 days, *n* (%)	3.3	2.9–3.8	<0.001	2.3	1.9–2.9	<0.001
ICU stay > 3 days, *n* (%)	4.9	4.3–5.4	<0.001	3.1	2.6–3.7	<0.001
Inhospital mortality, *n* (%)	4.6	3.0–7.0	<0.001	3.8	2.8–5.0	<0.001
1-year mortality, *n* (%)	3.1	2.4–3.9	<0.001	1.8	1.3–2.6	0.001
Long-term mortality, *n* (%)	3.0	2.6–3.4	<0.001	2.4	1.8–3.0	<0.001

*OR, odds ratio; 95% CI, 95% confidence interval*.

# *Adjusted for significant variables include age, sex, BMI, comorbidity (hypertension, chronic heart failure, and chronic kidney disease), blood gas results (pH value, base excess, anion gap, bicarbonate, and lactate), laboratory tests (white blood cell count, platelets count, hemoglobin, serum creatinine, and international normalized ratio), SOFA score, and SIRS score*.

The result showed that perioperative transfusion was associated with increased inhospital mortality (OR = 4.6, 95% CI 3.0–7.0), 1-year mortality (OR = 3.1, 95% CI 2.4–3.9), and long-term mortality (OR = 3.0, 95% CI 2.6–3.4) and also showed a significantly increased risk for the secondary outcomes.

Furthermore, after adjusting for the significant variables in baseline, inhospital mortality (OR = 3.8, 95% CI 2.8–5.0), 1-year mortality (OR = 1.8, 95% CI 1.3–2.6), and long-term mortality (OR = 2.4, 95% CI 1.8–3.0) and the secondary outcomes were significantly higher for the transfusion group (All *P* ≤ 0.001).

### Outcome Analysis

**Figure 1:**
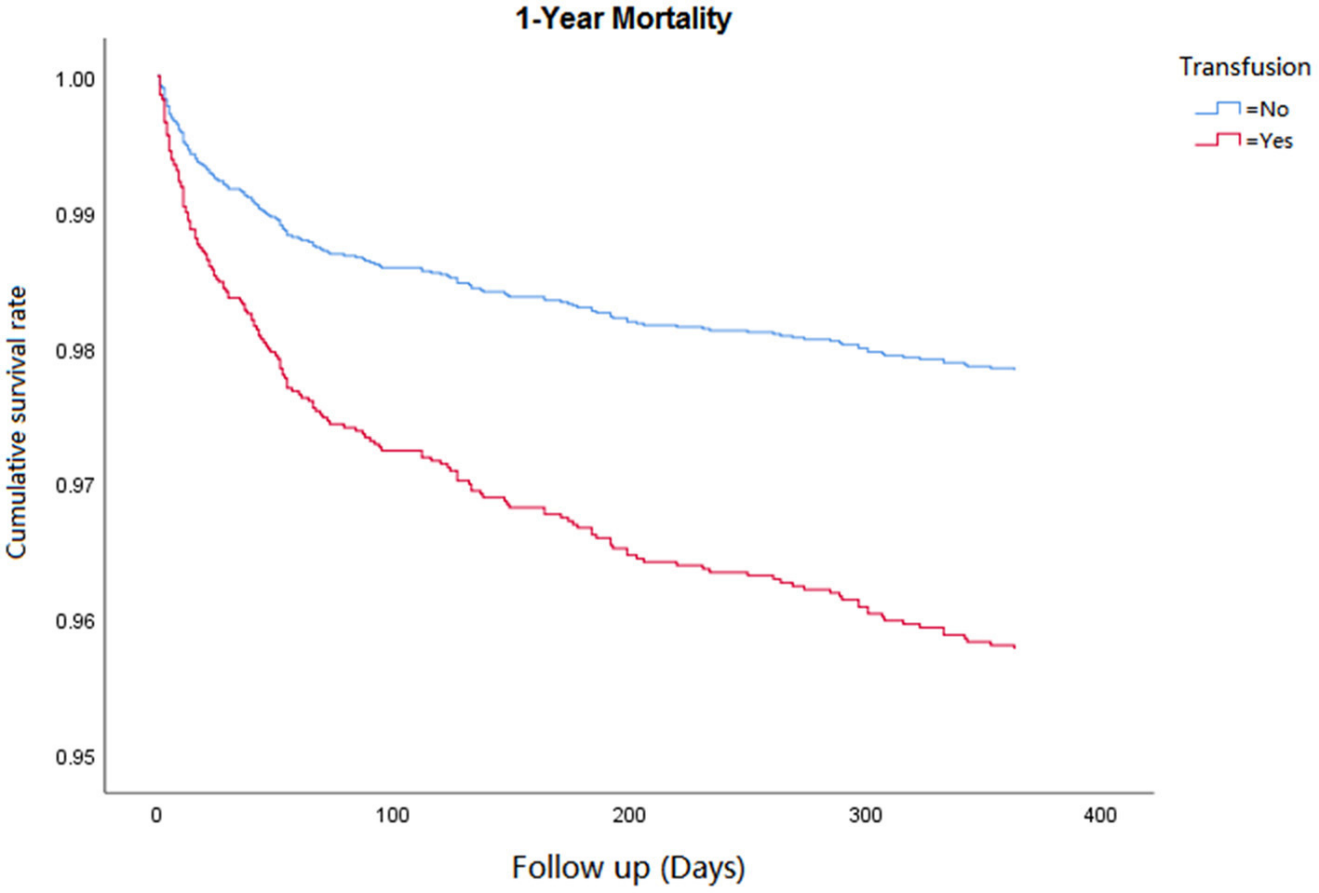
Correlation between perioperative red blood cell transfusion and 1-year mortality.

**Figure 2:**
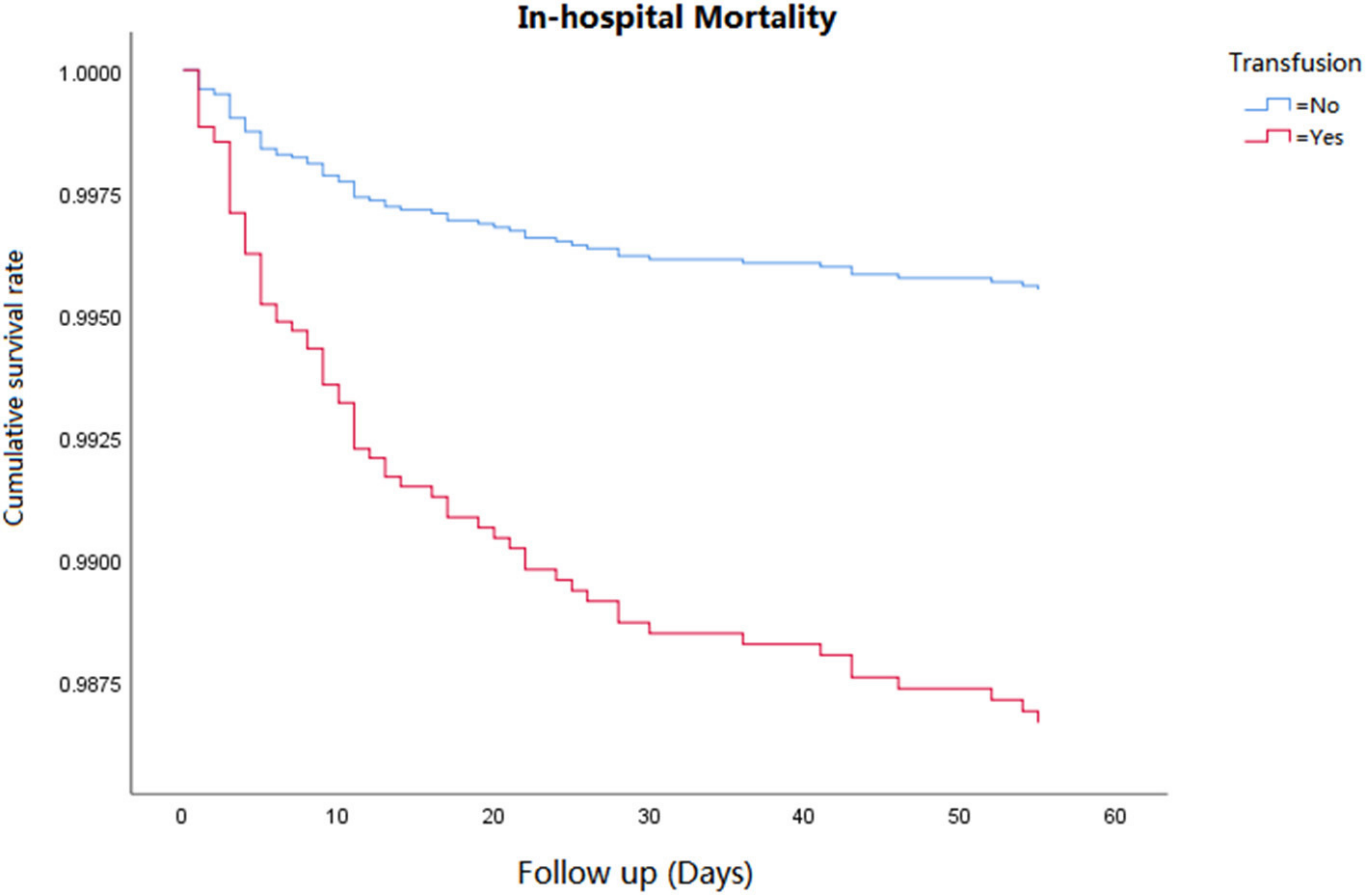
Correlation between perioperative red blood cell transfusion and inhospital mortality.

**Figure 3:**
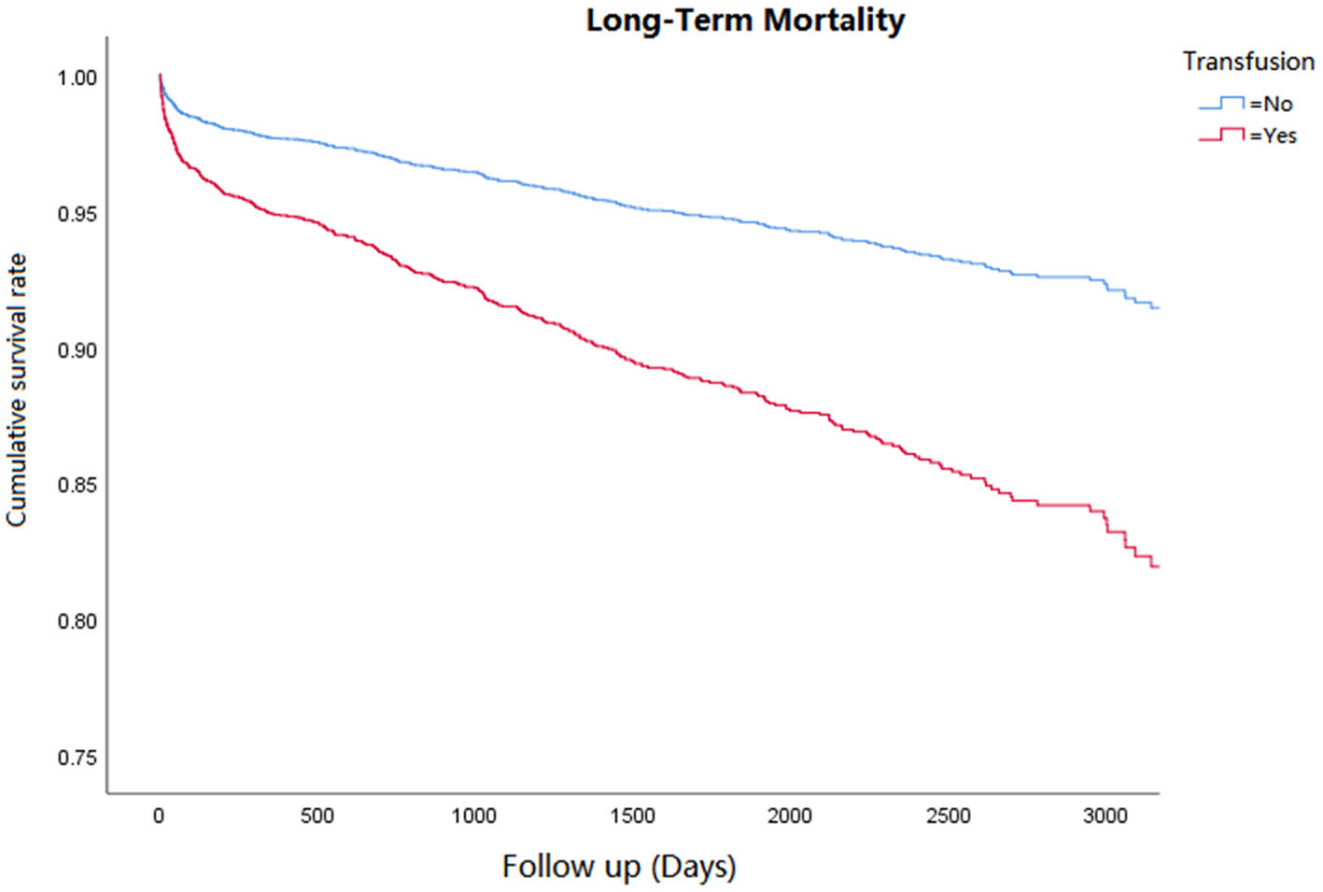
Correlation between perioperative red blood cell transfusion and long-term mortality.

Cox regression analysis was performed for the corresponding outcomes between groups (Table 3). After adjusting for the significant covariables, results illuminated that an increased risk of inhospital mortality (HR = 2.8, 95% CI 1.5–5.1, *P* = 0.001, Figure 1), 1-year mortality (HR = 2.0, 95% CI 1.4–2.7, *P* < 0.001, Figure 2), and long-term mortality (HR = 2.2, 95% CI 1.8–2.8, *P* < 0.001, Figure 3) were maintained in cardiac surgery patients with transfusion over those without transfusion.

**Table 3:** Cox multivariate regression model of association between perioperative red blood cell transfusion and postoperative outcomes.

	**Adjusted HR^#^**	**95% CI**	***P***
Inhospital mortality, *n* (%)	2.8	1.5–5.1	0.001
1-year mortality, *n* (%)	2.0	1.4–2.7	<0.001
Long-term mortality, *n* (%)	2.2	1.8–2.8	<0.001

*HR, hazard ratio; 95% CI, 95% confidence interval*.

# *Adjusted for significant variables include age, sex, BMI, comorbidity (hypertension, chronic heart failure, and chronic kidney disease), blood gas results (pH value, base excess, anion gap, bicarbonate, and lactate), laboratory tests (white blood cell count, platelets count, hemoglobin, serum creatinine, and international normalized ratio), SOFA score, and SIRS score*.

### Related Risk Factors for Perioperative Transfusion

The multivariate logistic regression analysis showed that age (OR = 1.01, 95% CI 1.00–1.02), CHF (OR = 1.51, 95% CI 1.33–1.80), diabetes mellitus (OR = 1.30, 95% CI 1.10–1.52), anion gap (AG) (OR = 1.06, 95% CI 1.03–1.09), lactate (OR = 1.11, 95% CI 1.06–1.18), SCr (OR = 1.20, 95% CI 1.12–1.34), INR (OR = 2.21, 95%CI 1.70–2.68), and SIRS score (OR = 1.32, 95%CI 1.23–1.46) were all associated with a significantly higher risk of perioperative RBC transfusion, whereas male gender (OR = 0.5, 95% CI 0.4–0.6), higher BMI (OR = 0.97; 95% CI 0.96–0.98), and base excess (BE) (OR = 0.95, 95% CI 0.92–0.98) were associated with a lower risk of transfusion (Table 4).

**Table 4:** Multivariate logistic regression analysis of the associated clinical characteristic.

	**OR**	**95% CI**	***P***
Age	1.01	1.00–1.02	0.002
Male	0.5	0.4–0.6	<0.001
BMI	0.97	0.96–0.98	<0.001
CHF	1.51	1.33–1.80	<0.001
DM	1.30	1.10–1.52	0.003
BE	0.95	0.92–0.98	<0.001
AG	1.06	1.03–1.09	<0.001
Lactate	1.11	1.06–1.18	<0.001
SCr	1.20	1.12–1.34	<0.001
INR	2.21	1.70–2.68	<0.001
SIRS score	1.32	1.23–1.46	<0.001

*OR, odds ratio; 95% CI, 95% confidence interval*.

*BMI, body mass index; CHF, chronic heart failure; DM, diabetes mellitus; BE, base excess; AG, anion gap; SCr, serum creatinine; INR, international normalized ratio; SIRS, systemic inflammatory response system*.

*Adjusted for corresponding significant variables include age, sex, BMI, comorbidity (hypertension, chronic heart failure, chronic and kidney disease), blood gas results (pH value, base excess, anion gap, bicarbonate, and lactate), laboratory tests (white blood cell count, platelets count, hemoglobin, serum creatinine, and international normalized ratio), SOFA score, and SIRS score*.

### Impact of Different Cardiac Surgery on the Outcome

The main types of surgery selected in the current study were coronary artery bypass graft (CABG, 58.6%) and valve replacement (VR, 26.4%), and CABG + VR patients accounted for 15% of the total population. Among them, the inhospital mortality rate of patients undergoing CABG + VR was 3%, which was significantly higher than solely CABG (1.4%) and VR (2.0%) patients. However, further logistics and Cox regression analysis showed no correlation between the type of surgery and patients' outcome. Including the type of surgery as a variable in the multivariate regression analysis had no significant effect on the correlation between RBC transfusion and clinical outcome indicators.

## Discussion

Our study demonstrated that perioperative RBC transfusion was associated with a worse prognosis of cardiac surgery patients. The primary outcomes—inhospital mortality, 1-year mortality, and long-term mortality—were all significantly higher in the cardiac surgery patients with transfusion. With further regression analysis, perioperative transfusions remained to be associated with an increased risk of the primary outcomes. In our study, patients of the transfusion group often presented with worse preoperative clinical conditions. They were significantly older, presented more frequently with CHF, were more likely to have metabolic disturbance (higher AG, lactate, and SCr), and have a higher SOFA score.

Perioperative management is a key component of cardiac surgery patients, especially for those with anemia. As an established risk factor reflecting systemic dysfunction and cardiovascular disease in the general population (14), anemia is a common comorbidity in perioperative patients (2, 4). It has been shown that the combination of anemia and blood transfusion carries additive risks for the incidence of adverse outcomes (15), and increasing units of RBC transfusion are associated with worse outcomes (16, 17). Previous studies showed that perioperative RBC transfusion in cardiac surgery could increase patients' risk of death, regardless of the amount of blood units (17–19).

Although the exact mechanism remains unclear, inflammation might be one of the significant factors. First, as our logistic regression analysis demonstrated, patients' systemic condition (age, sex, lower BMI, and comorbidity), metabolic status (BE, AG, lactate, SCr, and INR), and inflammation (SIRS Score) appear to affect the requirement of transfusion and their prognosis as well. Similar perioperative risk factors have been noted to have an inverse relationship to the prognosis in cardiac surgery (20). For example, studies showed a higher mortality risk for the underweight and the extremely obese patients after cardiac surgery (21). These factors reflect the general systemic condition of the internal environment and homeostasis, which might affect the outcomes and contribute to a higher risk of death (22–26). Therefore, optimal perioperative management should be considered, especially in these high-risk patients with multiple comorbidities and/or systemic status.

Second, despite RBC transfusions having significant values in anemic treatment, inadequate allogeneic transfusion may put patients at an additional risk of future cardiovascular events led by tissue ischemia, increased red-cell aggregation, and pro-inflammatory cytokines (27). Patients who require cardiac surgery usually have concomitant cardiac dysfunction with secondary complications, such as malnutrition. Pro-inflammatory pathways and cardiac inflammation would be activated for regulating tissue stress and/or malfunction (20). Also, factors related to the surgery (such as direct injury or CPB,) (1) have the potential to influence the postoperative course of systemic inflammatory response, leading to worse outcomes. Therefore, the vicious cycle of inflammation and cardiac dysfunction could be formed to affect patients' outcomes.

Due to the factors mentioned earlier, a restricted transfusion strategy with a lower HB trigger of blood transfusion has been proposed and shown to be non-inferior to liberal strategies in different clinical settings (28) or even with improved outcomes (29). Adopting the restrictive strategy in cardiac surgery, previous randomized trials have shown non-inferior 28-day and 6-month outcomes compared with liberal strategies as well (29–31). Our results regarding real-world settings showed a substantial impact on primary outcomes, which reinforced the unanswered questions of blood usage in cardiac surgery patients. Trials continue to integrate the optimal strategy, and assessing transfusion thresholds is needed. With the advancement of perioperative management for cardiac surgery, restrictive transfusion strategy with complementary methods [such as central venous oxygen saturation (32), near-infrared spectroscopy (33), etc.] might further optimize patient management.

Furthermore, various covariates might affect the decision of transfusion and outcomes in cardiac surgery patients, such as elective or emergency setting (34), type of CPB (on/off-pump), and perioperative anticoagulation (35). However, data regarding the factors mentioned earlier in the MIMIC database were not complete and therefore could not be used to evaluate the impact on transfusion and outcomes. Adjustments with coagulation-related indexes (platelet and INR) to minimize such bias were applied in the multivariate regression. Also, analysis with limited data suggested the type of cardiac surgery might account for the limited impact on outcomes.

In summary, our results showed that worse short-term and long-term outcomes were observed in patients receiving perioperative RBC transfusion. Although the association between these subsequent factors in management and prognosis has yet to be conclusively demonstrated, a worse preexisting systemic condition and/or the inflammatory response might be the possible mechanism. Comprehensive optimal management and close follow-up are recommended for cardiac surgery patients who needed perioperative transfusion according to the current guidelines. Also, further exploration of the restrictive transfusion strategy regarding the threshold and clinical setting is needed for optimal interventions and improving outcomes.

## Limitation

Although the present study demonstrated the association between the RBC transfusion and the outcomes of cardiac surgery patients, several limitations should be considered when interpreting these data. Firstly, although the sample size is relatively large, this was a retrospective study based on a single-center database. Secondly, anemia type and threshold for the transfusion were not further characterized due to the limitation of the dataset, which might limit the generalization of the results. Finally, it reports only preliminary results for clinical outcomes without control for covariables, such as surgery settings (CPB, on/off pump, and emergency/elective) and the use of anticoagulants. Further explorations regarding the correlation between postoperative systemic conditions, surgery settings, and transfusion threshold in adverse outcomes are needed.

## Conclusion

In this large retrospective analysis, we demonstrated that perioperative RBC transfusion might affect both the short-term and long-term prognoses of cardiac surgery patients. Results suggested that restrictive transfusion strategy might be further explored in cardiac surgery patients for optimal management. To be noted, patients with significant comorbidities were more likely to receive a blood transfusion, suggesting advanced perioperative management and follow-up might be needed in these selective patients.

## Data Availability Statement

Publicly available datasets were analyzed in this study. This data can be found here: https://mimic.mit.edu.

## Ethics Statement

Ethical review and approval was not required for the study on human participants in accordance with the local legislation and institutional requirements. The ethics committee waived the requirement of written informed consent for participation.

## Author Contributions

YL participated in the design of the research and drafted the manuscript. IC participated in the design and was a major contributor in writing the manuscript. ZZ participated in the analyses and interpretation of the study statistic design. XZ supervised the study program and method feasibility. QC and JL contributed conception and design of the research and performed critical revision of the manuscript for important intellectual content. All authors read and approved the final manuscript.

## Conflict of Interest

The authors declare that the research was conducted in the absence of any commercial or financial relationships that could be construed as a potential conflict of interest.

## Publisher's Note

All claims expressed in this article are solely those of the authors and do not necessarily represent those of their affiliated organizations, or those of the publisher, the editors and the reviewers. Any product that may be evaluated in this article, or claim that may be made by its manufacturer, is not guaranteed or endorsed by the publisher.
